# Effectiveness of RSV Vaccines against RSV-Associated Thromboembolic Events

**DOI:** 10.3201/eid3202.251520

**Published:** 2026-02

**Authors:** Ryan E. Wiegand, Heng-Ming Sung, Yue Zhang, Andrea Chavez, Amber Kautz, Josephine Mak, Morgan Najdowski, Yangping Chen, Yenlin Lai, Yixin Jiao, Yoganand Chillarige, Ruth Link-Gelles, Amanda B. Payne

**Affiliations:** Centers for Disease Control and Prevention, Atlanta, Georgia, USA (R.E. Wiegand, A. Kautz, J. Mak, M. Najdowski, R. Link-Gelles, A.B. Payne); Acumen LLC, Burlingame, California, USA (H.-M. Sung, Y. Zhang, A. Chavez, Y. Chen, Y. Lai, Y. Jiao, Y. Chillarige); General Dynamics Information Technology, Atlanta (A. Kautz); Eagle Health Analytics, San Antonio, Texas, USA (M. Najdowski); US Public Health Service Commissioned Corps, Rockville, Maryland, USA (R. Link-Gelles)

**Keywords:** respiratory syncytial virus, viruses, vaccine-preventable diseases, thromboembolism, vaccine effectiveness, United States

## Abstract

We evaluated effectiveness of respiratory syncytial virus (RSV) vaccines against RSV-associated thromboembolic events among community-dwelling Medicare fee-for-service beneficiaries >65 years of age in the United States enrolled during October 1, 2023–March 30, 2024. RSV vaccines protected against RSV-associated thromboembolic events (effectiveness 79% [95% CI 74%–83%]) in the same season as vaccine receipt.

Respiratory virus infections, including respiratory syncytial virus (RSV) infections, have been associated with increased risk for myocardial infarction (*1*), ischemic stroke (*2*), and venous thromboembolism (*3*). In 1 US-based surveillance network, ≈22% of adults >50 years of age who were hospitalized with RSV experienced an acute cardiac event (*4*).

In June 2023, the Advisory Committee on Immunization Practices recommended a single dose of RSV vaccine for adults >60 years of age to be determined on the basis of shared clinical decision making (*5*). RSV vaccines have reduced the likelihood of RSV-associated hospitalizations in immunocompetent and immunocompromised adults >60 years of age and have reduced RSV-associated emergency department visits in immunocompetent adults >60 years of age by 70%–80% (*6*). Our goal was to evaluate the effectiveness of a single dose of RSV vaccine against RSV-associated thromboembolic events in community-dwelling Medicare beneficiaries >65 years of age during the same season as RSV vaccine receipt. Understanding the effectiveness of RSV vaccines against RSV-associated thromboembolic events could guide policy makers, clinicians, and patients on how to reduce the risk for serious cardiovascular outcomes caused by RSV.

## The Study

Medicare fee-for-service beneficiaries >65 years of age on September 10, 2023 (index date), were eligible for inclusion in a retrospective cohort provided they met all inclusion and exclusion criteria (Appendix). Follow-up time began on October 1, 2023, and ended on the date when a beneficiary experienced an RSV-associated thromboembolic event, another censoring event (Appendix), or the end of study (March 30, 2024), whichever came first.

An RSV-associated thromboembolic event consisted of a myocardial infarction, ischemic stroke, or venous thromboembolism (Appendix Table 2) 7 days before to 30 days after an RSV diagnosis (Appendix Table 1). We identified RSV vaccine doses through Medicare Part D claims by using National Drug Code Directory codes (Appendix Table 3). A beneficiary was unvaccinated for RSV until they received an RSV vaccine dose and was vaccinated for RSV starting at 14 days after the RSV vaccine administration date. We excluded the period from vaccine receipt through day 13 after receipt.

Multivariable Cox proportional hazards models in R version 4.4.0 (The R Project for Statistical Computing, https://www.r-project.org) estimated vaccine effectiveness (VE) against RSV-associated thromboembolic events. RSV vaccination was a time-dependent covariate. The model adjusted results for age, sex, race/ethnicity, social vulnerability index (*7*) deciles, rural or urban location, immunocompromise status (Appendix Table 4), nonimmunocompromising underlying medical conditions (Appendix Table 5), previous season influenza vaccination (Appendix Table 6), and current season COVID-19 vaccination (Appendix Table 7). We stratified results by immunocompromise status (immunocompetent or immunocompromised), age group (65–74 and >75 years of age), time since vaccination (14–59, 60–119, or >120 days), and RSV vaccine product (Arexvy, GSK, https://www.gsk.com; and Abrysvo, Pfizer, https://www.pfizer.com). 

Sensitivity analyses consisted of an extended follow-up period for thromboembolic events through October 6, 2024; a follow-up limited to periods of high RSV circulation (defined as the period between 2 consecutive weeks >3% and 2 consecutive weeks <3% RSV prevalence) based on data from the National Respiratory and Enteric Virus Surveillance System (*8*); all-cause thromboembolic events, regardless of prior RSV diagnosis; and, to reduce residual confounding, models that incorporated inverse probability of treatment weights (IPTW). This activity was reviewed by the Centers for Disease Control and Prevention and deemed not to be research; it was conducted consistent with applicable federal law and agency policy per 45 CFR §46. This study presented minimal risk to participants because no patient interaction or intervention occurred; therefore, a waiver of informed consent was granted. This study followed the Strengthening the Reporting of Observational Studies in Epidemiology reporting guidelines (https://www.strobe-statement.org).

The analytic population consisted of 15,558,386 beneficiaries (Appendix Table 8); 58% (n = 8,998,133) were women, 80% (n = 12,376,268) were in an urban location, and 13% had immunocompromising conditions (Appendix Table 9). RSV VE against RSV-associated thromboembolic events was 79% (95% CI 74%–83%) for all beneficiaries (Table). VE estimates did not differ substantially between immunocompromised beneficiaries (VE 69% [95% CI 56%–78%]) and immunocompetent beneficiaries (VE 82% [95% CI 77%–86%]). Estimated VE among beneficiaries 65–74 years of age was 75% (95% CI 63%–83%), and estimated VE among beneficiaries >75 years of age was 80% (95% CI 74%–84%). VE point estimates by time since vaccination were all within 4 percentage points (14–59 days, VE 80% [95% CI 72%–86%]; 60–119 days, VE 79% [95% CI 72%–84%]; >120 days, VE 75% [95% CI 60%–84%]). Product-specific VE estimates did not differ substantially (Arexvy, VE 76% [95% CI 70%–81%]; Abrysvo, VE 85% [95% CI 77%–90%]).

**Table:** Adjusted VE of RSV vaccine against RSV-associated TEs among community-dwelling Medicare beneficiaries >65 years of age, United States, October 1, 2023–March 30, 2024*

Stratification or vaccination status	No. beneficiaries	No. RSV-associated TEs	Total no. TEs per 10,000 person-years	Median follow-up days contributed to category	Outcome rates per 10,000 person-years	Adjusted VE, % (95% CI)
Overall						
Unvaccinated	12,353,511	2,405	627	181	3.84	Referent
Vaccinated	3,204,875	96	109	132	0.88	79 (74–83)
Immunocompromised						
Unvaccinated	1,587,615	523	81	181	6.46	Referent
Vaccinated	509,928	36	17	131	2.07	69 (56–78)
Immunocompetent						
Unvaccinated	10,765,895	1,882	546	181	3.45	Referent
Vaccinated	2,694,947	60	92	132	0.65	82 (77–86)
Age 65–74 y						
Unvaccinated	6,711,712	630	341	181	1.85	Referent
Vaccinated	1,605,200	27	55	132	0.49	75 (63–83)
Age ≥75 y						
Unvaccinated	5,641,799	1,775	286	181	6.20	Referent
Vaccinated	1,599,675	69	54	131	1.27	80 (74–84)
Time since vaccination, d						
14–59	208,379	33	38	46	0.87	80 (72–86)
60–119	840,280	44	44	60	1.01	79 (72–84)
>120†	2,156,216	19	28	46	0.68	75 (60–84)
Vaccine product						
Arexvy‡	2,193,463	74	74	130	1.00	76 (70–81)
Abrysvo§	1,011,412	22	35	137	0.63	85 (77–90)

*Adjusted VE estimates from multivariable Cox proportional hazards models after controlling for age group, sex, race/ethnicity, social vulnerability index deciles, rural or urban category (determined by location of a beneficiary’s facility in a US Census Core Based Statistical Area or not), a count of the number of underlying medical conditions, immunocompromise status, influenza vaccination in the previous season, and COVID-19 vaccination during the current season. VE calculated by using the formula VE = (1 – hazard ratio) × 100 (Appendix, https://wwwnc.cdc.gov/EID/article/32/2/25-1520-App1.pdf). RSV, respiratory syncytial virus; TEs, thromboembolic events; VE, vaccine effectiveness. †Maximum number of days a beneficiary in interim analysis is contributing is 127 days. ‡GlaxoSmithKline, https://www.gsk.com. §Pfizer, https://www.pfizer.com.

Extending the follow-up period for thromboembolic events yielded VE estimates of 78% (95% CI 74%–82%), and limiting analyses to periods of high RSV circulation yielded VE estimates of 79% (95% CI 73%–83%) (Appendix Tables 10, 11). Estimates of RSV VE against all-cause thromboembolic events, regardless of prior RSV diagnosis, were lower (VE 21% [95% CI 19%–22%]) than for primary analyses (Appendix Table 12). VE against RSV-associated thromboembolic events based on models with IPTW was 71% (95% CI 62%–77%) (Appendix Table 13), which was not substantially different from the estimate obtained in models without IPTW (Table).

## Conclusions

Among a retrospective cohort of >15 million community-dwelling Medicare beneficiaries >65 years of age, RSV vaccines provided protection against RSV-associated thromboembolic events in the same season as RSV vaccination. Across all immunocompetent subgroups, VE estimates ranged from 75% to 85%; VE was 69% among immunocompromised beneficiaries. As expected, RSV vaccines provided higher protection against RSV-associated thromboembolic events compared with all-cause thromboembolic events.

This study demonstrates the effectiveness of RSV vaccines against RSV-associated thromboembolic events, including myocardial infarction, ischemic stroke, and venous thromboembolism. Our findings are consistent with studies demonstrating that influenza and COVID-19 vaccines reduce the likelihood of thromboembolic events in adults (*9*,*10*). Estimates from these analyses are comparable to other surveillance platforms that have estimated RSV VE against RSV-associated hospitalization (*6*,*11*). Time since vaccination results suggest minimal to no waning over the first 4 months postvaccination. Other analyses of RSV-associated hospitalization demonstrated more noticeable waning over a shorter period (*6*).

One limitation of these estimates are that Medicare beneficiaries with parts A, B, and D coverage might not be representative of the US population of adults >65 years of age. In addition, misclassification of RSV vaccination and RSV-associated outcomes are possible because both rely on administrative claims data. Vaccinations and outcome events not recorded in the claims data were not captured. The extent to which potential misclassification and under capture might have affected VE estimates is not clear. Although models adjusted for multiple covariates, residual confounding attributable to differences between the vaccinated and unvaccinated groups might still exist, especially in unmeasured confounders (e.g., smoking history). Our results indicate that VE against all-cause thromboembolic events was lower than VE against RSV-associated thromboembolic events but not 0%, which might suggest misclassification of the outcome or residual confounding. We did not have sufficient power to evaluate VE against the components of our definition of thromboembolic events.

In summary, we found that RSV vaccinations provided protection against RSV-associated thromboembolic events in adults >65 years of age in the same season as vaccine receipt. Protection was high regardless of immunocompromise status, age group, or RSV vaccine product. As of June 2025, RSV vaccine recommendations for adults in the United States have expanded to a single dose of RSV vaccine for adults 50–64 years of age with certain high-risk conditions and all adults >75 years of age (*12*,*13*).

Appendix Additional information about effectiveness of RSV vaccines against RSV-associated thromboembolic events.
