# Supplementary material for: Effectiveness of RSV Vaccines against RSV-Associated Thromboembolic Events
Source: Emerg Infect Dis. 2026 Feb;32(2):246–9. doi: 10.3201/eid3202.251520 (PMC12928231; doi:10.3201/eid3202.251520)
Supplement: Appendix — Additional information about effectiveness of RSV vaccines against RSV-associated thromboembolic events. [file 25-1520-Techapp-s1.pdf]

Article DOI: DOI: https://doi.org/10.3201/eid3202.251520

EID cannot ensure accessibility for supplementary materials supplied by authors. Readers who have difficulty accessing supplementary content should contact the authors for assistance.

Effectiveness of RSV Vaccines against RSV-Associated Thromboembolic Events

Appendix

Methods

Inclusion and exclusion criteria

Beneficiaries were eligible for inclusion on the index date if they were continuously enrolled in Medicare Part A/B (but not Part C or Medicare Advantage) for 365 days prior to the index date, and continuously enrolled in Part D starting from June 21, 2023.

Beneficiaries were excluded if they had a dialysis encounter, a nursing facility stay of ≥100 days, evidence of hospice care, an RSV diagnosis, or a thromboembolic event in the 365 days prior to the index date. Additional exclusions were an RSV vaccine prior to June 21, 2023 (indicating receipt of a dose during a clinical trial or a data entry error as RSV vaccines were not widely available prior to June 2023), a claim for a second dose of RSV vaccine >7 days after an initial vaccination, or any censoring event during the 14 days after vaccination (if vaccination was before the index date) or between the index date and the start of follow-up.

Censoring events

Other censoring events included instances where a beneficiary died, disenrolled from Medicare Parts A/B/D, enrolled into Medicare Part C, began hospice care, reached a nursing home stay of 100 consecutive days, had a dialysis encounter, or received a second RSV vaccine >7 days after the initial RSV vaccination, or occurrence of an outcome of interest or any censoring event <14 days after vaccination. Events during the first 13 days after vaccination were excluded because of the ambiguity of whether such an event should be attributed to the RSV unvaccinated group or the RSV vaccinated group.

RSV-associated thromboembolic event definition

Beneficiaries were recorded as having an RSV diagnosis if they had at least one facility claim or at least two professional service claims on distinct dates within 21 days listing a relevant International Classification of Diseases, Tenth Revision (ICD-10) code (Appendix Table 1).

Thromboembolic events were defined as the presence on an inpatient claim of a myocardial infarction or ischemic stroke diagnosis code or presence of a venous thromboembolism code recorded as present at admission with an associated procedure code within 7 days of the admission date (Appendix Table 2).

Subgroup and variable definitions

Immunocompromise was defined as a beneficiary with ≥2 encounters with a relevant ICD-10 code ≤183 days of the index date (Appendix Table 4). The number of non-immunocompromising underlying medical conditions was the number of condition groups recorded on claims ≤365 days of the index date (Appendix Table 5).

Influenza vaccination in the previous influenza season (August 1, 2022-June 30, 2023) was defined by presence of a claim listing a relevant Current Procedural Terminology (CPT) code (Appendix Table 6).

COVID-19 vaccination during the current season (September 10, 2023-March 30, 2024) was defined by presence of a claim listing a relevant CPT or Healthcare Common Procedure Coding System code (Appendix Table 7).

Details on statistical analysis

RSV vaccination status was treated as a time-dependent exposure. Thus, person time for the RSV vaccinated category is accumulated from beneficiaries 14 days or more after RSV vaccination and person time for the RSV unvaccinated group is accumulated from beneficiaries without an RSV vaccination

Hazard ratios (HRs) for time to first outcome of interest in our study period compared the survival curve of RSV-associated thromboembolic events among beneficiaries 14 days or more after RSV vaccination to the survival curve of RSV-associated thromboembolic events among beneficiaries without an RSV vaccination. Vaccine effectiveness (VE) was calculated using the formula:

$$\mathrm{VE} = (1 - HR) * 100.$$

VE estimates were adjusted by age group, sex, race/ethnicity, Social Vulnerability Index (*1*) deciles, rural/urban classification based on whether a beneficiary's facility was in a U.S. Census Core Based Statistical Area, the number of underlying medical conditions, immunocompromise status, influenza vaccination in the previous season, and COVID-19 vaccination during the current season.

Inverse probability of treatment weights (IPTW) sensitivity analyses

We performed a sensitivity analysis using inverse probability of treatment weights (IPTWs) for VE against an RSV-associated thromboembolic event.

A marginal Structural Cox regression model (Cox-MSM) was fit to estimate HRs among individuals in the RSV vaccinated cohort compared with individuals in the RSV non-vaccinated cohort. MSMs estimated the effect of the vaccination on the outcome by controlling for the time-dependent confounders by assigning a weight to each observation based on the inverse of their likelihood of receiving a particular treatment regimen.

Initial treatment weights were calculated using logistic regression to estimate a propensity score for each beneficiary. The propensity score quantified the likelihood of receiving an RSV vaccine dose prior to start of the follow-up period. Then, among unvaccinated beneficiaries at the start of follow-up, a time-varying treatment weight was calculated via a Cox proportional hazard model to estimate the propensity scores, which quantified the likelihood of receiving an RSV vaccine dose during the study period. Finally, a censoring weight was also estimated using a Cox proportional hazard model. The censoring weights quantified the likelihood of dropping out from the study for each beneficiary. Once those three quantities were estimated, original weights were computed as the inverse of the product of the initial treatment weights, the time-varying treatment weight, and the time-varying censoring weight (at 7-day intervals or when a beneficiary switched cohorts). Finally, the IPTW weight was created by truncating the original weight between 1% and 99% of the original weight.

In the IPTW adjusted vaccine effectiveness estimates, the models were adjusted for influenza and COVID-19 vaccine covariates.

We assessed covariate balance across cohorts using the standardized mean difference (SMD) of all covariates over time. SMD was defined as the difference in means (for continuous variables) or proportions (for categorical/binary variables) of a variable divided by the pooled standard deviation of the variable. An SMD of 0.1 or less indicated a negligible difference in means or proportions between groups.

Reference

1. Flanagan BE, Gregory EW, Hallisey EJ, Heitgerd JL, Lewis B. A social vulnerability index for disaster management. J Homel Secur Emerg Manag. 2011;8:3. https://doi.org/10.2202/1547-7355.1792

Appendix Table 1. RSV diagnosis codes, International Classification of Diseases, Tenth Revision

Code	Description
B974	Respiratory syncytial virus as the cause of diseases classified elsewhere
J121	RSV pneumonia
J210	Acute bronchiolitis due to RSV
J205	Acute bronchitis due to RSV

Appendix Table 2. Thromboembolic event codes, International Classification of Diseases, Tenth Revision, Clinical Modification (ICD-10-CM), International Classification of Diseases, Tenth Revision, Procedure Coding System (ICD-10-PCS), Healthcare Common Procedure Coding System (HCPCS) or Current Procedural Terminology (CPT)

Thromboembolic Event	Codes
Ischemic Stroke (ICD-10-CM)	G08, G43601, G43609, G43611, G43619, G450, G451, G452, G453, G454, G458, G489, I63, I630, I6300, I6301, I63011, I63012, I63013, I63019, I6302, I6303, I63031, I63032, I63033, I63039, I6309, I631, I6310, I6311, I63111, I63112, I63113, I63119, I6312, I6313, I63131, I63132, I63133, I63139, I6319, I632, I6320, I6321, I63211, I63212, I63213, I63219, I6322, I6323, I63231, I63232, I63233, I63239, I6329, I633, I6330, I6331, I63311, I63312, I63313, I63319, I6332, I63321, I63322, I63323, I63329, I6333, I63331, I63332, I63333, I63339, I6334, I63341, I63342, I63343, I63349, I6339, I634, I6340, I6341, I63411, I63412, I63413, I63419, I6342, I63421, I63422, I63423, I63429, I6343, I63431, I63432, I63433, I63439, I6344, I63441, I63442, I63443, I63449, I6349, I635, I6350, I6351, I63511, I63512, I63513, I63519, I6352, I63521, I63522, I63523, I63529, I6353, I63531, I63532, I63533, I63539, I6354, I63541, I63542, I63543, I63549, I6359, I636, I638, I6381, I6389, I639, I6501, I6502, I6503, I6509, I651, I6521, I6522, I6523, I6529, I658, I659, I6601, I6602, I6603, I6609, I6611, I6612, I6613, I6619, I6621, I6622, I6623, I6629, I663, I668, I669, I672, I676, I6782, I693, I6930, I6931, I69310, I69311, I69312, I69313, I69314, I69315, I69318, I69319, I6932, I69320, I69321, I69322, I69323, I69328, I6933, I69331, I69332, I69333, I69334, I69339, I6934, I69341, I69342, I69343, I69344, I69349, I6935, I69351, I69352, I69353, I69354, I69359, I6936, I69361, I69362, I69363, I69364, I69365, I69369, I6939, I69390, I69391, I69392, I69393, I69398, I6980, I97810, I97811, I97820, I97821, O225, O2250, O2251, O2252, O2253, O873, P910, R29700, R29701, R29702, R29703, R29704, R29705, R29706, R29707, R29708, R29709, R29710, R29711, R29712, R29713, R29714, R29715, R29716, R29717, R29718, R29719, R29720, R29721, R29722, R29723, R29724, R29725, R29726, R29727, R29728, R29729, R29730, R29731, R29732, R29733, R29734, R29735, R29736, R29737, R29738, R29739, R29740, R29741, R29742
Myocardial Infarction (ICD-10-CM)	I21, I210, I2101, I2102, I2109, I211, I2111, I2119, I212, I2121, I2129, I213, I214, I219, I21A, I21A1, I21A9, I22, I220, I221, I222, I228, I229, I23, I230, I231, I232, I233, I234, I235, I236, I237, I238, I24, I240, I241, I248, I249
Venous Thromboembolism (ICD-10-CM)	I8010, I260, I2601, I2602, I2609, I269, I2690, I2692, I2693, I2694, I2699, I8010, I8011, I8012, I8013, I80201, I80202, I80203, I80209, I80211, I80212, I80213, I80219, I80221, I80222, I80223, I80229, I80231, I80232, I80233, I80239, I80241, I80242, I80243, I80249, I80251, I80252, I80253, I80259, I80291, I80292, I80293, I80299, I803, I808, I809, I81, I820, I82210, I82220, I82290, I823, I82401, I82402, I82403, I82409, I82411, I82412, I82413, I82419, I82421, I82422, I82423, I82429, I82431, I82432, I82433, I82439, I82441, I82442, I82443, I82449, I82451, I82452, I82453, I82459, I82461, I82462, I82463, I82469, I82491, I82492, I82493, I82499, I824Y1, I824Y2, I824Y3, I824Y9, I824Z1, I824Z2, I824Z3, I824Z9, I82601, I82602, I82603, I82609, I82621, I82622, I82623, I82629, I82890, I8290, I82A11, I82A12, I82A13, I82A19, I82B11, I82B12, I82B13,

Thromboembolic Event	Codes
	I82B19, I82C11, I82C12, I82C13, I82C19, K550, O2230, O2231, O2232, O2233, O871, O88211, O88212, O88213, O88219, O8822, O8823
Venous Thromboembolism procedure code (HCPCS/CPT)	1522, 33310, 33315, 34401, 34421, 34451, 34471, 34490, 36005, 37187, 37188, 37191, 70548, 70549, 72191, 73206, 74174, 74175, 74185, 75820, 75822, 75825, 75827, 78445, 78455, 78456, 78457, 78458, 93965, 93970, 93971, 93978, 93979, R612626, 71275, 71550, 71551, 71552, 71555, 75741, 75743, 75746, 78580, 78582, 78597, 78598, 33910, 33915
Venous Thromboembolism procedure code (ICD-10-PCS)	6A750Z6, 6A751Z6, B22600Z, B2260ZZ, B22610Z, B2261ZZ, B226Y0Z, B226YZZ, B226ZZZ, B236Y0Z, B236YZZ, B236ZZZ, B24BZZ3, B24CZZ3, B30S0ZZ, B30S1ZZ, B30SYZZ, B30T0ZZ, B30T1ZZ, B30TYZZ, B31S0ZZ, B31S1ZZ, B31SYZZ, B31T0ZZ, B31T1ZZ, B31TYZZ, B31U0ZZ, B31U1ZZ, B31UYZZ, B340ZZ3, B341ZZ3, B342ZZ3, B34HZZ3, B34HZZZ, B34JZZ3, B34JZZZ, B34KZZ3, B34KZZZ, B34SZZ3, B34TZZ3, B44FZZ3, B44FZZZ, B44GZZ3, B44GZZZ, B44HZZ3, B44HZZZ, B44LZZ3, B546ZZ3, B547ZZ3, B548ZZ3, B549ZZ3, B54BZZ3, B54BZZZ, B54CZZ3, B54CZZZ, B54DZZ3, B54DZZZ, B54MZZ3, B54MZZZ, B54NZZ3, B54NZZZ, B54PZZ3, B54PZZZ, B835Y0Z, B835YZZ, B835ZZZ, B836Y0Z, B836YZZ, B836ZZZ, B837Y0Z, B837YZZ, B837ZZZ, BF35Y0Z, BF35YZZ, BF35ZZZ, BF36Y0Z, BF36YZZ, BF36ZZZ, BF37Y0Z, BF37YZZ, BF37ZZZ, BH3DY0Z, BH3DYZZ, BH3DZZZ, BP2W0ZZ, BP2W1ZZ, BP2WYZZ, BT31Y0Z, BT31YZZ, BT31ZZZ, BT32Y0Z, BT32YZZ, BT32ZZZ, BT33Y0Z, BT33YZZ, BT33ZZZ, BT39Y0Z, BT39YZZ, BT39ZZZ, BU4CZZZ, BV49ZZZ, BW03ZZZ, BW2400Z, BW240ZZ, BW2410Z, BW241ZZ, BW24Y0Z, BW24YZZ, BW24ZZZ, BW33Y0Z, BW33YZZ, BW38Y0Z, BW38YZZ, BW38ZZZ, BW3FY0Z, BW3FYZZ, BW3FZZZ, BW41ZZZ

Appendix Table 3. RSV vaccination codes, Vaccine Administration Codes (CVX) and National Drug Code (NDC) Directory

Brand / Manufacturer	CVX Code	NDC Code	Code Description	Manufacturer Description
Pfizer / Abrysvo	305	00069-0344-01	Respiratory syncytial virus vaccine, preF,	Vial and prefilled syringe presentation supplied in carton of 1 kit
		00069-0344-05	subunit, bivalent, for intramuscular use; via,	Vial and prefilled syringe presentation supplied in carton of 5 kits
		00069-0344-10	0.5 mL, reconstituted	Vial and prefilled syringe presentation supplied in carton of 10 kits
		00069-0207-01		Lyophilized Antigen Component, included in each kit and carton
		00069-2465-19	Respiratory syncytial	Sterile Water Diluent, included in Act-O-Vials
		00069-2465-01	virus vaccine, preF,	Act-O-Vial presentation supplied in carton of 1
		00069-2465-10	subunit, bivalent, for intramuscular use; vial, 0.5 mL, Single-dose	Act-O-Vial presentation supplied in carton of 10
		00069-0250-01	-	Prefilled syringe containing Sterile Water Diluent Component, included in each kit
		00069-0651-01	-	Vials of Sterile Water Diluent, included in each carton
		00069-1265-10	-	Vial and vial presentation supplied in cartons of 5
		00069-1265-20	-	Vial and vial presentation supplied in cartons of 10
GSK / Arexvy	303	58160-0848-11	Respiratory syncytial virus vaccine, preF, recombinant, subunit, adjuvanted; vial, 0.5 mL,	Outer carton; a single-dose vial of lyophilized antigen component (powder) and a single-dose vial of adjuvant suspension component (liquid) (packaged without syringes or needles)
		58160-0744-03	reconstituted	10 vials of lyophilized antigen (powder)
		58160-0723-03		10 vials of adjuvant suspension (liquid)

Appendix Table 4. Immunocompromising conditions (IC) codes, International Classification of Diseases, Tenth Revision (ICD-10)

Condition	ICD-10 Codes
Hematological Malignancy, likely IC status	C81.*, C82.*, C83.*, C84.*, C85.*, C86.*, C88.*, C90.*, C91.*, C92.*, C93.*, C94.*, C95.*, C96.*, D46.*, D61.0*, D70.0, D61.2, D61.9, D71.*
Other intrinsic immune condition or immunodeficiency, likely IC status	D27.9, D72.89, D80.*, D81.0, D81.1, D81.2, D81.4, D81.5, D81.6, D81.7, D81.8*, D81.9, D82.*, D83.*, D84.*, D89.0, D89.1, D89.3, D89.4*, D89.8*, D89.9, K70.3*, K70.4*, K72.*, K74.3, K74.4, K74.5, K74.6*, N04.*, R18.0
Solid Malignancy, likely IC status	C00.*, C01.*, C02.*, C03.*, C04.*, C05.*, C06.*, C07.*, C08.*, C09.*, C10.*, C11.*, C12.*, C13.*, C14.*, C15.*, C16.*, C17.*, C18.*, C19.*, C20.*, C21.*, C22.*, C23.*, C24.*, C25.*, C26.*, C30.*, C31.*, C32.*, C33.*, C34.*, C37.*, C38.*, C39.*, C40.*, C41.*, C43.*, C45.*, C46.*, C47.*, C48.*, C49.*, C50.*, C51.*, C52.*, C53.*, C54.*, C55.*, C56.*, C57.*, C58.*, C60.*, C61.*, C62.*, C63.*, C64.*, C65.*, C66.*, C67.*, C68.*, C69.*, C70.*, C71.*, C72.*, C73.*, C74.*, C75.*, C76.*, C77.*, C78.*, C79.*, C7A.*, C7B.*, C80.*, Z51.0, Z51.1*, C4A.*
Transplant, likely IC status	T86.0*, T86.1*, T86.2*, T86.3*, T86.4*, T86.5*, T86.81*, T86.85*, D47.Z1, Z48.2*, Z94.*, Z98.85
Rheumatologic/inflammatory disorders, likely IC status	D86.*, E85.1, E85.2, E85.3, E85.4, E85.8*, E85.9, G35.*, J67.9*, L40.54, L40.59, L93.0*, L93.2*, L94.*, M05.*, M06.*, M07.*, M08.*, M30.*, M31.3*, M31.5*, M32.*, M33.*, M34.*, M35.3*, M35.89, M35.9*, M46.0*, M46.1, M46.8*, M46.9*
HIV, possible IC status	B20.*, B21.*, B22.*, B23.*, B24.*, B97.35, O98.7*, Z21.*

Appendix Table 5. Non-immunocompromising underlying medical condition codes, International Classification of Diseases, Tenth Revision (ICD-10)

Condition Group	Condition	ICD-10 Codes
Lung Disease	Asthma	J45.*
Lung Disease	COPD	J40.*, J41.*, J42.*, J43.*, J44.*
Lung Disease	Other Chronic Lung Disease	D86.0, E88.01, J47.*, J60, J61, J62.*, J63.*, J64, J65, J66.*, J67.0, J67.1, J67.2, J67.3, J67.4, J67.5, J67.6, J67.7, J67.8, J68.*, J70.*, J81.1, J84.*, J95*, J96.1*, J99.*, P26.*, P27.*, B39.*, B40.0, B40.1, B40.2, B41.0, B44.0, B44.1, B45.*, B46.0, A15.*, A31.0
Lung Disease	Cystic Fibrosis	E84.*
Cardiovascular Disease	Heart Failure	I50.*
Cardiovascular Disease	Ischemic Heart Disease	I21.*, I22.*, I23.*, I24.*, I25.*
Cardiovascular Disease	Other Heart Disease	I01.*, I02.0, I09.*, I27.*, I28.*, I31.*, I42.*, I43.*, I44.*, I46.*, I51.0, I51.1, I51.2, I51.3, I51.5, I51.7, I51.8*, I51.9, I52.*, I97.0, I97.1*, M31.0, M31.1*, M31.2, M31.4, M31.6, M31.7, M31.8, M31.9, Z95.*, Z98.61, I71.*, I72.*, I73.*, I74.*, I75.*, I79.*
Cardiovascular Disease	Pulmonary Embolism	I26.*
Cardiovascular Disease	Heart valve disorders	I05.*, I06.*, I07.*, I08.*, I34.*, I35.*, I36.*, I37.*
Cardiovascular Disease	Atrial fibrillation and flutter	I48.*
Cardiovascular Disease	Congenital heart disease	I50.9, I42.9, Q20.*, Q21.*, Q22.*, Q23.*, Q24.*, Q25.*, Q26.*, Q27.0, Q27.3*, Q27.4, Q27.8, Q27.9, Q28.*, Q33.*, Q89.3, P29.30
Diabetes	Diabetes associated with organ damage	E08.2*, E08.3*, E08.4*, E08.5*, E08.610, E09.2*, E09.3*, E09.4*, E09.5*, E09.610, E10.2*, E10.3*, E10.4*, E10.5*, E10.610, E11.2*, E11.3*, E11.4*, E11.5*, E11.610, E13.2*, E13.3*, E13.4*, E13.5*, E13.610
Neurologic	Dementia (including Alzheimer's)	F01.*, F02.*, F03.*, G30.*
Neurologic	Muscular dystrophy	G71.0*
Neurologic	Down's Syndrome	Q90.*
Liver Disorder	Liver Disease	B18.*, I81.*, I85.*, K70.*, K71.*, K72.*, K73.*, K74.*, K75.*, K76.*, K77.*
Hematologic Disorders	Blood Disorder	D55.*, D56.0, D56.1, D56.2, D56.4, D56.5, D56.8, D56.9, D57.0*, D57.1, D57.2*, D57.4*, D57.8*, D58.*, D59.*, D60.*, D61.*, D64.0, D64.1, D64.2, D64.3, D64.4, D64.8*, D65.*, D66.*, D67.*, D68.*
Obesity	Clinical Obesity	E66.*, Z68.3*, Z68.4*

Appendix Table 6. Influenza vaccination codes, Current Procedural Terminology (CPT)

CPT Code	Description
90662	Vaccine for influenza for injection into muscle, split virus, enhanced immunogenicity via increased antigen content
90672	Vaccine for influenza for nasal administration, tetravalent
90674	Vaccine for influenza for administration into muscle, 0.5 ml dosage, tetravalent, cell-culture based
90682	Influenza virus vaccine, quadrivalent (RIV4), derived from recombinant DNA, hemagglutinin (HA) protein only, preservative and antibiotic free, for intramuscular use)
90686	Vaccine for influenza for administration into muscle, 0.5 ml dosage, quadrivalent, preservative free
90687	Vaccine for influenza for administration into muscle, 0.25 ml dosage, quadrivalent (pediatric use)
90688	Vaccine for influenza for administration into muscle, 0.5 ml dosage, quadrivalent
90694	Influenza virus vaccine, quadrivalent (allV4), inactivated, adjuvanted, preservative free, 0.5 mL dosage, for intramuscular use
90756	Influenza virus vaccine, quadrivalent (ccIIV4), derived from cell cultures, subunit, antibiotic free, 0.5mL dosage, for intramuscular use)
90630	Influenza virus vaccine, quadrivalent (IIV4), split virus, preservative free, for intradermal use
90658	Influenza virus vaccine, trivalent (IIV3), split virus, 0.5 mL dosage, for intramuscular use
90657	Influenza virus vaccine, trivalent (IIV3), split virus, 0.25 mL dosage, for intramuscular use
90656	Influenza virus vaccine, trivalent (IIV3), split virus, preservative free, 0.5 mL dosage, for intramuscular use
90653	Influenza vaccine, inactivated (IIV), subunit, adjuvanted, for intramuscular use
90673	Influenza virus vaccine, trivalent (RIV3), derived from recombinant DNA, hemagglutinin (HA) protein only, preservative and antibiotic free, for intramuscular use
90661	Influenza virus vaccine, trivalent (ccIIV3), derived from cell cultures, subunit, antibiotic free, 0.5 mL dosage, for intramuscular use
90662	Influenza virus vaccine (IIV), split virus, preservative free, enhanced immunogenicity via increased antigen content, for intramuscular use
90660	Influenza virus vaccine, trivalent, live (LAIV3), for intranasal use
90470	H1N1 immunization administration (intramuscular, intranasal), including counseling when performed
90654	Influenza virus vaccine, trivalent (IIV3), split virus, preservative-free, for intradermal use
90659	Influenza virus vaccine, whole virus, for intramuscular or jet injection use
90663	Influenza virus vaccine, pandemic formulation, H1N1
90664	Influenza virus vaccine, live (LAIV), pandemic formulation, for intranasal use
90666	Influenza virus vaccine (IIV), pandemic formulation, split virus, preservative free, for intramuscular use
90668	Influenza virus vaccine (IIV), pandemic formulation, split virus, for intramuscular use
90685	Influenza virus vaccine, quadrivalent (IIV4), split virus, preservative free, 0.25 mL dosage, for intramuscular use
90727	Influenza virus vaccine

Appendix Table 7. COVID-19 vaccination codes, Healthcare Common Procedure Coding System (HCPCS) or Current Procedural Terminology (CPT)

Brand	Code Type	CPT Code	Vaccine Administration Code
Pfizer	HCPCS/CPT	91300	0001A (1st dose)<br>0002A (2nd dose)<br>0003A (3rd dose)<br>0004A (Booster)
	HCPCS/CPT	91305	0051A (1st dose)<br>0052A (2nd dose)<br>0053A (3rd dose)<br>0054A (Booster)
	HCPCS/CPT	91312	0124A (Booster)
	HCPCS/CPT	91318	90480
	HCPCS/CPT	91319	90480
	HCPCS/CPT	91320	90480
Moderna	HCPCS/CPT	91301	0011A (1st dose)<br>0012A (2nd dose)<br>0013A (3rd dose)
	HCPCS/CPT	91306	0064A (Booster)
	HCPCS/CPT	91309	0094A (Booster)
	HCPCS/CPT	91313	0134A (Booster)
	HCPCS/CPT	91321	90480
	HCPCS/CPT	91322	90480
Janssen	HCPCS/CPT	91303	0031A (Single dose)<br>0034A (Booster)
Novavax	HCPCS/CPT	91304	0041A (1st dose)<br>0042A (2nd dose)<br>0044A (Booster)
Other	CPT	M0201<br>91302	Not Applicable

Appendix Table 8. Exclusions for the retrospective cohort study of RSV vaccine effectiveness against thromboembolic events among Medicare beneficiaries in the United States aged ≥65 years, October 1, 2023, to March 30, 2024

	Criteria	Count of Beneficiaries	Count of Beneficiaries Excluded From Previous Row	Proportion of Beneficiaries Excluded from Previous Row	Cumulative Proportion of Beneficiaries Excluded from Total
Base Population					
A	Alive and aged 65 or older on index date *(Season 1 index date: September 10, 2023)*	59,832,445	-	-	-
B	Satisfying (A) and being continuously enrolled in Medicare Part A/B, and not C, for 365 days prior to index date	23,248,240	36,584,205	61.1%	61.1%
C	Satisfying (B) and being continuously enrolled in Medicare Part D starting from June 21, 2023	16,657,399	6,590,841	28.3%	72.2%
D	Satisfying (C) and having no evidence of dialysis encounter during the 365 days prior to index date	16,577,439	79,960	0.5%	72.3%
E	Satisfying (D) and having no evidence of nursing home/skilled nursing facility stay for ≥100 days at time of index date within 365 days prior to index date	16,219,618	357,821	2.2%	72.9%
F	Satisfying (E) and having no evidence of hospice care within 365 days prior to index date	16,100,452	119,166	0.7%	73.1%
G	Satisfying (F) and having no evidence of RSV diagnosis within 365 days prior to the index date	16,072,684	27,768	0.2%	73.1%
H	Satisfying (G) and having no evidence of any thromboembolic events within 365 days prior to the index date	15,628,542	444,142	2.8%	73.9%
I	Satisfying (H) and having no evidence of receiving RSV vaccine prior to June 21, 2023	15,628,542	0	0.0%	73.9%
J	Satisfying (I) and having no evidence of receiving a second dose of RSV vaccine 7 days or more after the initial vaccination	15,628,507	35	0.0%	73.9%
K	Satisfying (J) and having no evidence of any outcome of interest or censoring event during the 14 days after vaccination, if original vaccination date is before index date	15,628,424	83	0.0%	73.9%
Population for RSV VE against RSV-associated thromboembolic events (primary analysis)					
L	Satisfying (K) and having no evidence of any outcome of interest or censoring event during the time between index date and start of follow-up window (October 1, 2023 for season 1) (i.e., left censoring)	15,558,386	70,038	0.4%	74.0%

Appendix Table 9. Summary statistics and demographic information for the retrospective cohort study of RSV vaccine effectiveness against RSV-associated thromboembolic events (TEs) among Medicare beneficiaries in the U.S. aged ≥65 years, October 1, 2023, to March 30, 2024

Covariate	Overall Beneficiaries (n, %)	Unvaccinated Beneficiaries (n, %)	Vaccinated Beneficiaries (n, %)	Standardized Mean Difference†
Total RSV-Associated TE Outcome Eligible Population	15,558,386	12,353,511	3,204,875	
Demographic and Socioeconomic Factors				
Age (Continuous)				
Median (IQR)	*74 (70, 79)*	*74 (69, 79)*	*74 (70, 79)*	
Age Group				
65-74	8,316,912 (53)	6,711,712 (54)	1,605,200 (50)	0.09
75+	7,241,474 (47)	5,641,799 (46)	1,599,675 (50)	0.09
Sex				
Men	6,560,253 (42)	5,203,800 (42)	1,356,453 (42)	0.00
Women	8,998,133 (58)	7,149,711 (58)	1,848,422 (58)	0.00
Social Vulnerability Index (SVI)				
1 ≤ SVI < 10	2,122,367 (14)	1,578,097 (13)	544,270 (17)	0.12
10 ≤ SVI < 20	2,081,284 (13)	1,576,626 (13)	504,658 (16)	0.09
20 ≤ SVI < 30	1,959,114 (13)	1,514,094 (12)	445,020 (14)	0.05
30 ≤ SVI < 40	1,820,340 (12)	1,429,993 (12)	390,347 (12)	0.02
40 ≤ SVI < 50	1,692,338 (11)	1,348,636 (11)	343,702 (11)	0.01
50 ≤ SVI < 60	1,552,081 (10)	1,257,764 (10)	294,317 (9)	0.03
60 ≤ SVI < 70	1,361,804 (9)	1,122,425 (9)	239,379 (7)	0.06
70 ≤ SVI < 80	1,160,458 (7)	970,392 (8)	190,066 (6)	0.08
80 ≤ SVI < 90	943,434 (6)	803,304 (7)	140,130 (4)	0.09
90 ≤ SVI ≤ 100	699,862 (4)	614,382 (5)	85,480 (3)	0.12
Missing	165,304 (1)	137,798 (1)	27,506 (1)	0.03
State/Region‡				
Region 1	927,351 (6)	702,760 (6)	224,591 (7)	0.05
Region 2	1,445,221 (9)	1,200,115 (10)	245,106 (8)	0.07
Region 3	1,727,488 (11)	1,363,763 (11)	363,725 (11)	0.01
Region 4	3,093,589 (20)	2,515,013 (20)	578,576 (18)	0.06
Region 5	2,513,304 (16)	1,966,210 (16)	547,094 (17)	0.03
Region 6	1,592,699 (10)	1,304,407 (11)	288,292 (9)	0.05
Region 7	908,070 (6)	704,567 (6)	203,503 (6)	0.03
Region 8	582,757 (4)	443,996 (4)	138,761 (4)	0.04
Region 9	2,084,037 (13)	1,636,899 (13)	447,138 (14)	0.02
Region 10	680,150 (4)	512,243 (4)	167,907 (5)	0.05
Missing	3,720 (0)	3,538 (0)	182 (0)	0.02
Rural/Urban Classification*				
Rural	3,182,118 (20)	2,669,409 (22)	512,709 (16)	0.14
Urban	12,376,268 (80)	9,684,102 (78)	2,692,166 (84)	0.14
Non-Immunocompromising Underlying Medical Conditions				
Number of Conditions				
0	5,373,420 (35)	4,405,988 (36)	967,432 (30)	0.12
1	5,001,801 (32)	3,927,317 (32)	1,074,484 (34)	0.04
2	3,185,941 (20)	2,470,414 (20)	715,527 (22)	0.06
3	1,426,972 (9)	1,104,016 (9)	322,956 (10)	0.04
4	460,381 (3)	358,412 (3)	101,969 (3)	0.02
5	97,124 (1)	76,843 (1)	20,281 (1)	0.00
6	12,104 (0)	9,961 (0)	2,143 (0)	0.01
7	643 (0)	560 (0)	83 (0)	0.00
Lung Disease				
Any Lung Disease	3,110,071 (20)	2,323,920 (19)	786,151 (25)	0.14
Asthma	1,219,468 (8)	865,709 (7)	353,759 (11)	0.14
Chronic Obstructive Pulmonary Disease (COPD)	1,922,642 (12)	1,471,038 (12)	451,604 (14)	0.06
Cystic Fibrosis	1,843 (0)	1,235 (0)	608 (0)	0.01
Other Lung Disease	760,737 (5)	555,217 (4)	205,520 (6)	0.08
Cardiovascular Disease				
Any Cardiovascular Disease	6,724,224 (43)	5,232,061 (42)	1,492,163 (47)	0.08
Heart Failure	1,435,714 (9)	1,150,215 (9)	285,499 (9)	0.01
Ischemic Heart Disease	3,475,675 (22)	2,704,324 (22)	771,351 (24)	0.05
Pulmonary Embolism	157,973 (1)	121,885 (1)	36,088 (1)	0.01
Heart Valve Disorders	2,225,120 (14)	1,706,444 (14)	518,676 (16)	0.07
Atrial fibrillation and flutter	2,149,772 (14)	1,672,689 (14)	477,083 (15)	0.04
Congenital Heart Disease (CHD)	1,078,191 (7)	860,613 (7)	217,578 (7)	0.01
Other Cardiovascular Disease	4,050,779 (26)	3,161,185 (26)	889,594 (28)	0.05
Endocrine/Metabolic Disease				

Covariate	Overall Beneficiaries (n, %)	Unvaccinated Beneficiaries (n, %)	Vaccinated Beneficiaries (n, %)	Standardized Mean Difference†
Diabetes Associated with Organ Damage	2,037,293 (13)	1,620,195 (13)	417,098 (13)	0.00
Neurological Disease				
Any Neurological and Musculoskeletal Disease	633,669 (4)	544,179 (4)	89,490 (3)	0.09
Dementia (including Alzheimer's)	628,468 (4)	540,017 (4)	88,451 (3)	0.09
Muscular dystrophy	4,383 (0)	3,440 (0)	943 (0)	0.00
Down's Syndrome	1,527 (0)	1,350 (0)	177 (0)	0.01
Hepatic Disease				
Liver Disease	1,086,216 (7)	847,798 (7)	238,418 (7)	0.02
Hematologic Disorders				
Any Hematologic Disorder	620,400 (4)	478,887 (4)	141,513 (4)	0.03
Blood disorder	620,400 (4)	478,887 (4)	141,513 (4)	0.03
Sickle cell disease	17,877 (0)	14,384 (0)	3,493 (0)	0.00
Obesity				
Clinical Obesity	3,846,995 (25)	3,014,702 (24)	832,293 (26)	0.04
Immunocompromising Conditions**				
Any Immunocompromising Conditions	2,097,543 (13)	1,587,615 (13)	509,928 (16)	0.09
Other Vaccinations				
Influenza Vaccination Status in Previous Season				
Received 2022/2023 Influenza Vaccine	9,694,044 (62)	6,813,305 (55)	2,880,739 (90)	0.84
COVID-19 Vaccination Status During Season***				
Received 2023/2024 COVID-19 Vaccine	5,585,024 (36)	3,057,980 (25)	2,527,044 (79)	1.29

† An SMD of 0.1 or less indicated a negligible difference in means or proportions between groups. Alternatively, an SMD > 0.1 suggested the variable may need to be included in multivariable models to reduce confounding.
‡ State is defined by the facility ZIP code at the beneficiary's index date, then categorized into the associated U.S. Department of Health and Human Services Region, specifically: Region 1 including Connecticut, Maine, Massachusetts, New Hampshire, Rhode Island; Region 2 including New Jersey, New York, Puerto Rico, Virgin Islands; Region 3 including Delaware, District of Columbia, Maryland, Pennsylvania, Virginia, West Virginia; Region 4 including Alabama, Florida, Georgia, Kentucky, Mississippi, North Carolina, South Carolina, Tennessee; Region 5 including Illinois, Indiana, Michigan, Minnesota, Ohio, Wisconsin; Region 6 including Arkansas, Louisiana, New Mexico, Oklahoma, Texas; Region 7 including Iowa, Kansas, Missouri, Nebraska; Region 8 including Colorado, Montana, North Dakota, South Dakota, Utah, Wyoming; Region 9 including Arizona, California, Hawaii, Nevada, American Samoa, Commonwealth of the Northern Mariana Islands, Federated States of Micronesia, Guam, Marshall Islands, Republic of Palau; and Region 10 including Alaska, Idaho, Oregon, Washington.
* Beneficiaries were classified as urban if the beneficiary's facility ZIP code at index date was included in a U.S. Census Core Based Statistical Area. Rural classification included missing values.
** Requiring ≥2 encounters within 183 days from index date
*** Time-varying vaccination status determined on censoring date (with outcome of interest)

Appendix Table 10. Adjusted vaccine effectiveness (VE) of RSV vaccine against RSV-associated thromboembolic events among community-dwelling Medicare beneficiaries in the United States aged ≥65 years, October 1, 2023 -- March 30, 2024, with thromboembolic event follow-up through October 6, 2024

Stratification/Vaccination Status	No. of beneficiaries	No. of RSV-associated thromboembolic events	Total no. of 10,000 person-years	Median follow-up days contributed to category	Outcome Rates (per 10,000 person-years)	Adjusted VE (95% CI)
Overall						
Unvaccinated (Ref)	12,353,544	3,491	627	181	5.57	*Ref*
Vaccinated	3,204,814	140	109	132	1.28	78% (74%, 82%)
Immunocompromised						
Unvaccinated (Ref)	1,587,631	820	81	181	10.12	*Ref*
Vaccinated	509,906	49	17	131	2.82	72% (63%, 79%)
Immunocompetent						
Unvaccinated (Ref)	10,765,913	2,671	546	181	4.89	*Ref*
Vaccinated	2,694,908	91	92	132	0.99	81% (76%, 84%)
Age 65-74 years						
Unvaccinated (Ref)	6,711,722	960	341	181	2.82	*Ref*
Vaccinated	1,605,180	36	55	132	0.65	78% (69%, 84%)
Age ≥ 75 years						
Unvaccinated (Ref)	5,641,822	2,531	286	181	8.85	*Ref*
Vaccinated	1,599,634	104	54	131	1.91	78% (74%, 82%)
Time (Days) Since Vaccination						
14-59 days since vaccination	208,370	47	38	46	1.23	81% (74%, 86%)
60-119 days since vaccination	840,267	66	44	60	1.51	78% (71%, 83%)

Stratification/Vaccination Status	No. of beneficiaries	No. of RSV-associated thromboembolic events	Total no. of 10,000 person-years	Median follow-up days contributed to category	Outcome Rates (per 10,000 person-years)	Adjusted VE (95% CI)
≥120 days since vaccination*	2,156,177	27	28	46	0.97	73% (60%, 82%)
Vaccine Product Brand						
Arexvy by GSK vaccinated	2,193,420	106	74	130	1.43	76% (70%, 80%)
Abrysvo by Pfizer vaccinated	1,011,394	34	35	137	0.97	83% (77%, 88%)

**Maximum number of days a beneficiary in interim analysis is contributing is 127.*

Notes: Adjusted vaccine effectiveness (VE) estimates from multivariable Cox proportional hazards models after controlling for age group, sex, race/ethnicity, Social Vulnerability Index deciles, rural/urban category determined by the location of a beneficiary's facility in a U.S. Census Core Based Statistical Area or not, a count of the number underlying medical conditions, immunocompromise status, influenza vaccination in the previous season, and COVID-19 vaccination during the current season. VE is calculated using the formula $VE = (1 - HR) * 100$.

Appendix Table 11. Adjusted vaccine effectiveness (VE) of RSV vaccine against RSV-associated thromboembolic events among community-dwelling Medicare beneficiaries in the United States aged ≥65 years, October 1, 2023 -- March 30, 2024, during high periods of RSV circulation by region†

Stratification/Vaccination Status	No. of beneficiaries	No. of RSV-associated thromboembolic events	Total no. of 10,000 person-years	Median follow-up days contributed to category	Outcome Rates (per 10,000 person-years)	Adjusted VE (95% CI)
Overall						
Unvaccinated (Ref)	12,509,795	2,100	463	132	4.53	*Ref*
Vaccinated	3,044,518	81	75	93	1.08	79% (73%, 83%)
Immunocompromised						
Unvaccinated (Ref)	1,611,539	433	60	118	7.24	*Ref*
Vaccinated	483,253	30	12	92	2.54	67% (53%, 78%)
Immunocompetent						
Unvaccinated (Ref)	10,898,256	1,667	404	132	4.13	*Ref*
Vaccinated	2,561,265	51	63	94	0.81	82% (76%, 87%)
Age 65-74 years						
Unvaccinated (Ref)	6,791,865	547	252	132	2.17	*Ref*
Vaccinated	1,528,118	24	38	95	0.63	73% (60%, 82%)
Age ≥ 75 years						
Unvaccinated (Ref)	5,717,930	1,553	211	132	7.36	*Ref*
Vaccinated	1,516,400	57	37	92	1.54	80% (74%, 85%)
Time (Days) Since Vaccination						
14-59 days since vaccination	516,069	31	32	46	0.96	80% (71%, 86%)
60-119 days since vaccination	1,238,406	39	32	60	1.21	78% (70%, 84%)
≥120 days since vaccination*	1,290,043	11	11	27	1.03	77% (59%, 88%)
Vaccine Product Brand						
Arexvy by GSK vaccinated	2,079,091	61	50	90	1.21	76% (69%, 82%)
Abrysvo by Pfizer vaccinated	965,427	20	25	101	0.80	84% (75%, 90%)

†High circulation periods were based on data from the National Respiratory and Enteric Virus Surveillance System (https://www.cdc.gov/nrevss/php/dashboard/index.html) and begin on October 14, 2023 (region 1), October 28, 2023 (region 2), October 7, 2023 (region 3), September 23, 2023 (region 4), October 21, 2023 (region 5), September 9, 2023 (region 6), October 28, 2023 (region 7), November 18, 2023 (region 8), September 30, 2023 (region 9), and October 28, 2023 (region 10).

*Maximum number of days a beneficiary in interim analysis is contributing is 127.

Notes: Adjusted vaccine effectiveness (VE) estimates from multivariable Cox proportional hazards models after controlling for age group, sex, race/ethnicity, Social Vulnerability Index deciles, rural/urban category determined by the location of a beneficiary's facility in a U.S. Census Core Based Statistical Area or not, a count of the number underlying medical conditions, immunocompromise status, influenza vaccination in the previous season, and COVID-19 vaccination during the current season. VE is calculated using the formula $VE = (1 - HR) * 100$.

Appendix Table 12. Adjusted vaccine effectiveness (VE) of RSV vaccine against all-cause thromboembolic events among community-dwelling Medicare beneficiaries in the United States aged ≥65 years, October 1, 2023 -- March 30, 2024

Stratification/Vaccination Status	No. of beneficiaries	No. of RSV-associated thromboembolic events	Total no. of 10,000 person-years	Median follow-up days contributed to category	Outcome Rates (per 10,000 person-years)	Adjusted VE (95% CI)
Overall						
Unvaccinated (Ref)	12,337,877	208,215	622	181	334.93	*Ref*
Vaccinated	3,193,211	26,180	109	131	240.78	21% (19%, 22%)
Immunocompromised						
Unvaccinated (Ref)	1,584,389	40,067	80	181	500.56	*Ref*
Vaccinated	507,302	6,150	17	131	357.02	18% (15%, 20%)
Immunocompetent						
Unvaccinated (Ref)	10,753,488	168,148	542	181	310.46	*Ref*
Vaccinated	2,685,909	20,030	92	132	218.90	21% (20%, 23%)
Age 65-74 years						
Unvaccinated (Ref)	6,706,361	70,383	339	181	207.76	*Ref*
Vaccinated	1,601,407	8,034	55	132	146.66	19% (17%, 21%)
Age ≥ 75 years						
Unvaccinated (Ref)	5,631,516	137,832	283	181	487.24	*Ref*
Vaccinated	1,591,804	18,146	54	131	336.37	21% (20%, 22%)
Time (Days) Since Vaccination						
14-59 days since vaccination	213,490	8,771	38	46	231.34	24% (22%, 26%)
60-119 days since vaccination	840,321	10,659	43	60	246.18	19% (18%, 21%)
≥120 days since vaccination*	2,139,400	6,750	28	46	245.30	17% (15%, 19%)
Vaccine Product Brand						
Arexvy by GSK vaccinated	2,185,575	17,563	74	130	237.91	22% (20%, 23%)
Abrysvo by Pfizer vaccinated	1,007,636	8,617	35	136	246.86	18% (17%, 20%)

*Maximum number of days a beneficiary in interim analysis is contributing is 127.

Notes: Adjusted vaccine effectiveness (VE) estimates from multivariable Cox proportional hazards models after controlling for age group, sex, race/ethnicity, Social Vulnerability Index deciles, rural/urban category determined by the location of a beneficiary's facility in a U.S. Census Core Based Statistical Area or not, a count of the number underlying medical conditions, immunocompromise status, influenza vaccination in the previous season, and COVID-19 vaccination during the current season. VE is calculated using the formula $VE = (1 - HR) * 100$.

Appendix Table 13. Adjusted vaccine effectiveness (VE) of RSV vaccine against RSV-associated thromboembolic events using inverse probability of treatment weights (IPTW) among community-dwelling Medicare beneficiaries in the United States aged ≥65 years, October 1, 2023 -- March 30, 2024.

Stratification/Vaccination Status	No. of beneficiaries	No. of RSV-associated thromboembolic events	Total no. of 10,000 person-years	Median follow-up days contributed to category	Outcome Rates (per 10,000 person-years)	Adjusted VE (95% CI)
Overall						
Unvaccinated (Ref)	12,353,511	2,405	627	181	3.84	Ref
Vaccinated	3,204,875	96	109	132	0.88	71% (62%, 77%)
Immunocompromised						
Unvaccinated (Ref)	1,587,615	523	81	181	6.46	Ref
Vaccinated	509,928	36	17	131	2.07	63% (44%, 75%)
Immunocompetent						
Unvaccinated (Ref)	10,765,896	1,882	546	181	3.45	Ref
Vaccinated	2,694,947	60	92	132	0.65	74% (63%, 81%)
Age 65-74 years						
Unvaccinated (Ref)	6,711,712	630	341	181	1.85	Ref
Vaccinated	1,605,200	27	55	132	0.49	66% (44%, 79%)
Age ≥ 75 years						

Stratification/Vaccination Status	No. of beneficiaries	No. of RSV-associated thromboembolic events	Total no. of 10,000 person-years	Median follow-up days contributed to category	Outcome Rates (per 10,000 person-years)	Adjusted VE (95% CI)
Unvaccinated (Ref)	5,641,799	1,775	286	181	6.20	Ref
Vaccinated	1,599,675	69	54	131	1.27	73% (63%, 80%)
Time (Days) Since Vaccination						
14-59 days since vaccination	208,379	33	38	46	0.87	76% (63%, 85%)
60-119 days since vaccination	840,280	44	44	60	1.01	70% (56%, 79%)
≥120 days since vaccination*	2,156,216	19	28	46	0.68	61% (31%, 78%)
Vaccine Product Brand						
Arexvy by GSK vaccinated	2,193,463	74	74	130	1.00	67% (56%, 75%)
Abrysvo by Pfizer vaccinated	1,011,412	22	35	137	0.63	79% (64%, 88%)

*Maximum number of days a beneficiary in interim analysis is contributing is 127.

Notes: Adjusted vaccine effectiveness (VE) estimates from multivariable Cox proportional hazards models after controlling for age group, sex, race/ethnicity, Social Vulnerability Index deciles, rural/urban category determined by the location of a beneficiary's facility in a U.S. Census Core Based Statistical Area or not, a count of the number underlying medical conditions, immunocompromise status, influenza vaccination in the previous season, and COVID-19 vaccination during the current season. VE is calculated using the formula $\mathrm{VE} = (1 - \mathrm{HR}) * 100$.
